# MR susceptibility contrast imaging using a 2D simultaneous multi-slice gradient-echo sequence at 7T

**DOI:** 10.1371/journal.pone.0219705

**Published:** 2019-07-17

**Authors:** Wei Bian, Adam B. Kerr, Eric Tranvinh, Sherveen Parivash, Benjamin Zahneisen, May H. Han, Christopher B. Lock, Maged Goubran, Kongrong Zhu, Brian K. Rutt, Michael M. Zeineh

**Affiliations:** 1 Department of Biomedical Engineering, School of Life Science and Technology, University of Electronic Science and Technology of China, Chengdu, China; 2 Department of Electrical Engineering, Stanford University, Palo Alto, CA, United States of America; 3 Department of Radiology, Stanford University, Palo Alto, CA, United States of America; 4 Department of Neurology and Neurological Sciences, Stanford University School of Medicine, Palo Alto, CA, United States of America; Wayne State University, UNITED STATES

## Abstract

**Purpose:**

To develop a 7T simultaneous multi-slice (SMS) 2D gradient-echo sequence for susceptibility contrast imaging, and to compare its quality to 3D imaging.

**Methods:**

A frequency modulated and phase cycled RF pulse was designed to simultaneously excite multiple slices in multi-echo 2D gradient-echo imaging. The imaging parameters were chosen to generate images with susceptibility contrast, including T_2_*-weighted magnitude/phase images, susceptibility-weighted images and quantitative susceptibility/R_2_* maps. To compare their image quality with 3D gradient-echo imaging, both 2D and 3D imaging were performed on 11 healthy volunteers and 4 patients with multiple sclerosis (MS). The signal to noise ratio (SNR) in gray and white matter and their contrast to noise ratio (CNR) was simulated for the 2D and 3D magnitude images using parameters from the imaging. The experimental SNRs and CNRs were measured in gray/white matter and deep gray matter structures on magnitude, phase, R_2_* and QSM images from volunteers and the visibility of MS lesions on these images from patients was visually rated. All SNRs and CNRs were compared between the 2D and 3D imaging using a paired t-test.

**Results:**

Although the 3D magnitude images still had significantly higher SNRs (by 13.0~17.6%), the 2D magnitude and QSM images generated significantly higher gray/white matter or globus pallidus/putamen contrast (by 13.3~87.5%) and significantly higher MS lesion contrast (by 5.9~17.3%).

**Conclusion:**

2D SMS gradient-echo imaging can serve as an alternative to often used 3D imaging to obtain susceptibility-contrast-weighted images, with an advantage of providing better image contrast and MS lesion sensitivity.

## Introduction

Gradient echo (GRE) sequences are frequently used in clinical MRI to provide images with magnetic susceptibility contrast, such as T_2_*-weighted, phase [1–4] and susceptibility-weighted images (SWI) [5–7], R_2_* mapping and quantitative susceptibility mapping (QSM) [4, 8–10]. These imaging methods are sensitive to myelin in white matter and iron in deep gray matter, making them especially useful in the evaluation of neurodegenerative and inflammatory diseases such as Alzheimer’s disease, Parkinson’s disease and multiple sclerosis (MS) [11–14]. Since susceptibility contrast scales with the magnetic field strength and high field scanners are becoming more widely available, high resolution GRE imaging is being pursued to better characterize normal anatomy and pathology [2,4,15].

Higher resolution GRE imaging is usually performed with a 3D sequence, which can achieve thin slices while maintaining sufficient signal to noise ratio (SNR) by exciting the whole imaging volume for each readout. In contrast, when the same imaging volume is scanned using a 2D sequence, the resulting images are often thought to have a lower SNR as the sequence excites only a single thin slice from the volume for each readout. However, it has been previously demonstrated that using interleaved multi-planar acquisition, 2D imaging can achieve comparable SNR to 3D imaging with a similar acquisition time [16]. This is because an interleaved acquisition allows 2D imaging to use a much longer TR and accordingly a much large flip angle than 3D imaging, hence compensating the SNR loss of 2D imaging due to its thin-slice excitation. However, the previous study [16] predated parallel imaging, which makes the SNR comparison difficulty at first inspection, because 2D parallel imaging has most commonly been accelerated only within a slice but not through slices, whereas 3D parallel imaging has been accelerated in both directions. This situation has changed with the advent of simultaneous multi-slice (SMS) imaging technique [17–19], as 2D imaging can now be accelerated both within and through slice.

The SMS technique was initially invented for 2D echo-planar imaging (EPI) to accelerate functional MRI and diffusion weighted MRI [17–19]. More recently, its application has been extended to non-EPI 2D spin-echo to increase turbo spin-echo imaging speed [20,21] and GRE imaging to achieve increased slice coverage in dynamic susceptibility contrast imaging [20], faster or larger coverage cardiac imaging [22–24], faster T_1_, T_2_, and proton density mapping [25], and banding reduction in balanced steady state free precession imaging [26]. However, no other attempts to our knowledge have been made to apply this technique to 2D intrinsic tissue susceptibility contrast imaging, nor to compare its image quality to 3D GRE imaging, when both of them can be accelerated through-slice as well as in-plane.

The purpose of this study is to develop a 2D multi-echo SMS GRE sequence at 7T to perform high-resolution T_2_*-weighted magnitude/phase imaging, SWI, R_2_* mapping and QSM, and compare their SNR and/or CNR to that obtained from a built-in product 3D GRE sequence with matched scan time, volume coverage and resolution.

## Theory

To do a theoretical comparison, the SNRs of 2D SMS and 3D GRE imaging were calculated using the following equations [27]

$$SNR_{2D}\propto S_{2D}\cdot \Delta x\Delta y\Delta z\sqrt{N_{x}N_{y}\Delta t}$$ (1)

and

$$SNR_{3D}\propto S_{3D}\cdot \Delta x\Delta y\Delta z\sqrt{N_{x}N_{y}N_{z}\Delta t}$$ (2)

respectively, where Δ*x*, Δ*y* and Δ*z* are the acquired voxel size, *N*_*x*_, *N*_*y*_, and *N*_*z*_ are number of acquired voxels, Δ*t* is the reciprocal of the readout bandwidth, and *S*_*2D*_ and *S*_*3D*_ are the signal intensity of 2D and 3D imaging, respectively. When a spoiled recalled gradient echo (SPGR) sequence is used, the signal intensity at the steady state is

$$S=\rho \frac{\left(1-exp\left(-TR/T_{1}\right)\right)\sin\alpha }{1-exp\left(-TR/T_{1}\right)\cos\alpha }exp\left(-TE/T_{2}^{*}\right)$$ (3)

Where *ρ* is the proton density. Assuming the same voxel size and Δ*t* for both 2D and 3D imaging and replacing *S*_*2D*_ and *S*_*3D*_ in Eqs (1) and (2) with Eq (3), the SNR ratio between the 3D and 2D is

$$\begin{array}{l} \frac{SNR_{3D}}{SNR_{{}_{2}D}}=\sqrt{N_{Z}}\cdot \frac{exp(-TE_{3D}/T_{2}^{*})}{exp(-TE_{2D}/T_{2}^{*})}\cdot \\ \frac{\left((1-exp\left(-TR_{3D}/T_{1}\right))\sin\alpha_{3D}\right)\left(1-exp\left(-TR_{2D}/T_{1}\right)\cos\alpha_{2D}\right)}{\left(\left(1-exp\left(-TR_{2D}/T_{1}\right)\right)\sin\alpha_{2D}\right)\left(1-exp\left(-TR_{3D}/T_{1}\right)\cos\alpha_{3D}\right)} \end{array}$$ (4)

When parallel imaging is performed, the imaging SNR will be penalized by a factor of the square root of the acceleration factor *R* and an additional factor of coil geometry *g*. On the other hand, the SNR of 2D SMS will be penalized only by the coil geometry factor but not the acceleration factor, because in SMS acquisitions phase encoding lines in k-space of any slice are fully sampled [17]. Therefore, when both parallel imaging and SMS are performed, Eq 4 becomes

$$\begin{array}{l} \frac{SNR_{3D}}{SNR_{{}_{2}D}}=\sqrt{\frac{N_{z}R_{2D_{inPlane}}}{R_{3D_{inPlane}}R_{3D_{slice}}}}\cdot \frac{g_{2D}}{g_{3D}}\cdot \frac{exp(-TE_{3D}/T_{2}^{*})}{exp(-TE_{2D}/T_{2}^{*})}\cdot \\ \frac{\left((1-exp\left(-TR_{3D}/T_{1}\right))\sin\alpha_{3D}\right)\left(1-exp\left(-TR_{2D}/T_{1}\right)\cos\alpha_{2D}\right)}{\left(\left(1-exp\left(-TR_{2D}/T_{1}\right)\right)\sin\alpha_{2D}\right)\left(1-exp\left(-TR_{3D}/T_{1}\right)\cos\alpha_{3D}\right)} \end{array}$$ (5)

where *R*_*2D*_*/g*_*2D*_ and *R*_*3D*_*/g*_*3D*_ are the parallel imaging acceleration (in-plane or through-slice)/coil geometry factors of 2D and 3D imaging, respectively. For a fair comparison, the acquisition time for 2D and 3D imaging needs to be same, which can be achieved by setting TR_*2D*_ to be Nz times of TR_*3D*_.

When SNRs from two different types of tissues are available and the noise is measured from the same region, the contrast to noise ratio (CNR) between the tissue *a* and *b* can be calculated by [28]

$$CNR=\left|SNR_{a}-SNR_{b}\right|$$ (6)

## Method

### Simulation

We calculated the SNR ratios in both white and gray matter using Eq 5 based on parameters used in our actual imaging sequences, with TE set to be the average of TEs of the multiple echoes, *R*_*3D_inPlane*_ = 3, *R*_*3D_slice*_ = 2, *R*_*2D_inPlane*_ = 2, SMS factor = 3, and tissue relaxation values measured in previous studies [29,30], where white matter T_1_ = 1126ms, T_2_* = 26.8ms and the gray matter T_1_ = 1939ms, T_2_* = 33.2ms. To simplify the simulation, the coil geometry factor was assumed to be equal for both 2D and 3D imaging. The ratio of 3D to 2D CNR between gray and white matter was also calculated according to Eq 6, with a factor of 1.2 being scaled to gray matter SNR, which is the proton density ratio between gray and white matter [31].

### Sequence design

**Fig 1:**
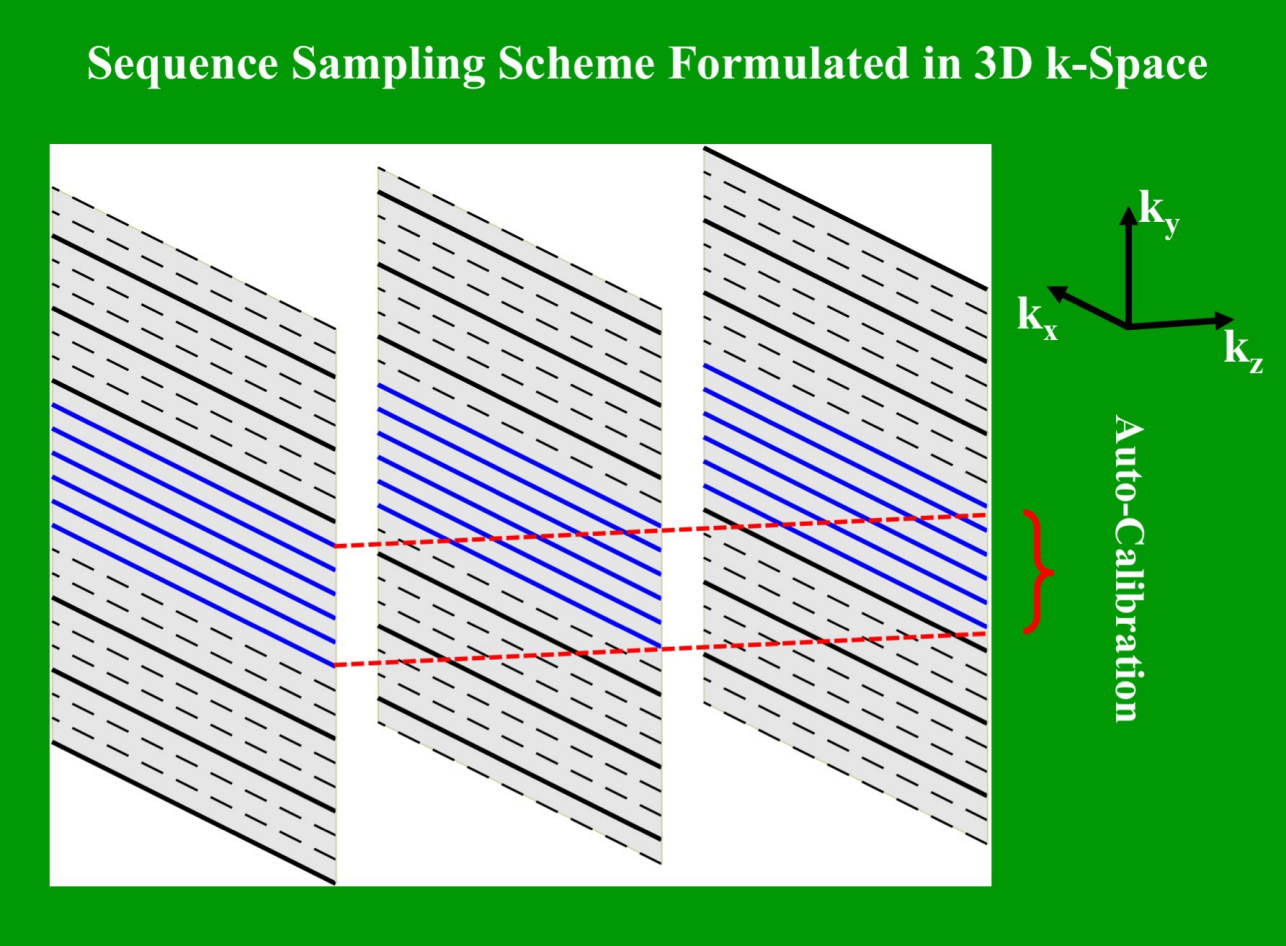
An illustration of the sampling scheme of the 2D SMS sequence formulated in the 3D k-space. In this 3-slice simultaneous excitation example, each solid line indicates a k-space line sampled after an RF excitation, which is also phase cycled to shift FOV among slices. The blue lines represent the auto-calibration region. In our study, only 16 calibrated lines in the center of the k-space were sampled to separate simultaneously acquired slices.

The RF pulse for the 2D SMS imaging was designed using the variable-rate selective excitation (VERSE) technique [32] to reduce the peak amplitude of the pulse (this modulation of the peak amplitude did not occur with SMS R = 3 in the experiments described here, but we chose VERSE to potentially accommodate higher SMS factors). During a multi-slice acquisition, this pulse was frequency modulated and then summed in real time, depending on the number of simultaneously excited slices, the distance between these slices, and the slice thickness. RF phase cycling was also applied to shift the FOV of N simultaneously excited slices by an amount of n/N (n = 0,1,…N-1) in order to reduce slice overlap and increase the SNR of images, using the technique termed controlled aliasing in parallel imaging results in higher acceleration (CAIPIRINHA) [33]. To provide a calibration for the SMS image reconstruction, an auto-calibration without a separate acquisition was performed by acquiring a few central k-space lines for each simultaneously excited slice. At the same time, these calibration lines were also used to calibrate the in-plane parallel imaging (Fig 1). To enable multi-echo imaging, multiple readout gradients were added, with the rest of the sequence components being identical to the typical SPGR sequence. To prevent potential cross-talk in SMS imaging when a slice band has an even number of slices [19], where an imperfect slice profile may saturate overlapping slices, a center-out slice ordering was implemented with the imaging starting from the center slice in a slice band and then spreading out on both sides.

### Subjects

Eleven healthy volunteers (5 females, mean age 41.0±14.1 years) and 4 patients with MS (1 female, mean age 42.8±12.1 years) were recruited (with their clinical information in Table 1). The study was approved by the Stanford University Institutional Review Board with the approval number of 00000935, and written informed consent was obtained from all participating subjects in accordance with the Health Insurance Portability and Accountability Act.

**Table 1:** Clinical information of multiple sclerosis patients.

Patient	Sex	Age	MS Type	Disease Duration	EDSS
**1**	Male	30	RR	1 year	3
**2**	Female	58	SP	31 years	7
**3**	Male	37	RR	1 year	2
**4**	Male	46	RR	16 years	6

EDSS = Estimated Disability Status Scale; RR = Relapse Remitting; SP = Secondary Progressive

### MR imaging

All imaging was performed on a GE Discovery 950 7T scanner (GE Healthcare, Waukesha, WI) with a 32-channel head coil (Nova Medical, Wilmington, MA). Both flow-compensated 2D and 3D SPGR imaging were performed using the following same parameters: FOV = 22cm, resolution = 0.5x0.5x1.5mm^3^, slice number = 84 (2 more slices were sampled at each end of the slab for 3D imaging), bandwidth = 41.7KHz, echo number = 4, and echo spacing = 7.1ms. The TEs, TR and flip angle (the Ernst angles of gray matter computed based upon measured T_1_s in the previous studies [29,30] and TRs used in our imaging, in order to optimize cortical SNR) of the 2D imaging were 8.2/15.3/22.4/29.5ms, 1050ms, and 54°, while those of 3D were 6.2/13.3/20.4/27.5ms, 33.6ms, and 11°. The 3D sequence was a built-in product sequence, which was thought to be optimal, hence no in-house modification was made. Because of this, the differences in the duration of RF pulse and slice selection/spoiling gradients between our 2D and the built-in 3D sequence resulted in their slightly different TEs and the 2D TR being a bit longer than the slice number times the 3D TR divided by the SMS factor. However, even with this increased TR, the total acquisition time of the 2D imaging was still made similar to that of the 3D imaging by choosing an appropriate number of parallel imaging calibration lines, causing no significant bias when comparing their SNRs. Three slices were simultaneously excited in the 2D imaging sequence with 16 auto-calibration lines. In-plane parallel imaging with an acceleration factor of 2 and 32 auto-calibration lines was also applied. In the 3D imaging, parallel imaging was applied both in-plane and through-slice, with an acceleration factor/auto-calibration lines of 3/24 and 2/24, respectively. Both sequences were acquired axially with the read-out direction being from anterior to the posterior. The total acquisition time was 4.75 minutes for 2D and 5.1 minutes for 3D imaging.

### Image reconstruction and analysis

For 2D SMS imaging, overlapped simultaneously-exited image slices were separated and reconstructed using the Slice-GRAPPA algorithm [17], in which the slice GRAPPA kernels were fitted using the data from the 16 auto-calibrated k-space lines. Before that, the aliased images due to in-plane acceleration were firstly recovered using the auto-calibrating reconstruction for Cartesian imaging (ARC) algorithm [34]. The same algorithm was also used to reconstruct both in-plane and through-plane accelerated 3D images using GE’s Orchestra platform. Magnitude images from each coil were combined using the root mean square method, while phase images from each coil were first unwrapped using the Laplacian method [9], then background removed using the projection onto dipole fields (PDF) method [35]. The phase images from each coil were finally combined voxel by voxel using a weighted average, where the weighing for each voxel from each coil was the voxel’s intensity on the magnitude image from the same coil. The above processing was repeated echo by echo to generate magnitude and phase images for each individual echo. The magnitude images from each echo were used to fit R_2_* maps using the nonlinear Levenberg-Marquardt method. The magnitude and phase images from each echo were averaged respectively to generate final magnitude and phase images [36], which were further processed to make SWI images using the method proposed by Haacke at el [6]. The phase images were also deconvolved using the morphological enhanced dipole inversion (MEDI) method to obtain QSM images [37].

### Data analysis

Regions of interest (ROIs) from putamen (Pu), globus pallidus (GP), internal capsules (IC), and homogeneous cingulate gray matter (GM) and white matter (WM) were manually defined on four consecutive axial slices on phase images, respectively for 2D and 3D images, by a medical student (SP) with 5 years of imaging research. The SNR was calculated in the GM and WM on magnitude images only, by dividing the mean value by the standard deviation in each region, respectively. The contrast to noise ratios (CNR) of the GP to Pu, GP to IC, and GM to WM were calculated for all imaging modalities by dividing the absolute difference in mean values between the two ROIs by the standard deviation in the IC (for the basal ganglia comparisons) or WM (for the GM to WM comparison). The visibility of MS lesions on all image modalities was rated by a neuroradiologist (ET) with 5 years of post-residency experience, who was blinded to the image acquisition type (i.e. 2D or 3D), using a four-level rating scale, with 1 being *poor* (not visible or barely visible), 2 being *fair* (faintly visible with limited contrast), 3 being *good* (visible with good contrast), and 4 being *excellent* (clearly visible with strong contrast). The SNRs, CNRs and lesion visibility between the 2D and 3D imaging were compared using a paired t-test with an uncorrected significance level of *p*<0.05.

### Results

**Fig 2:**
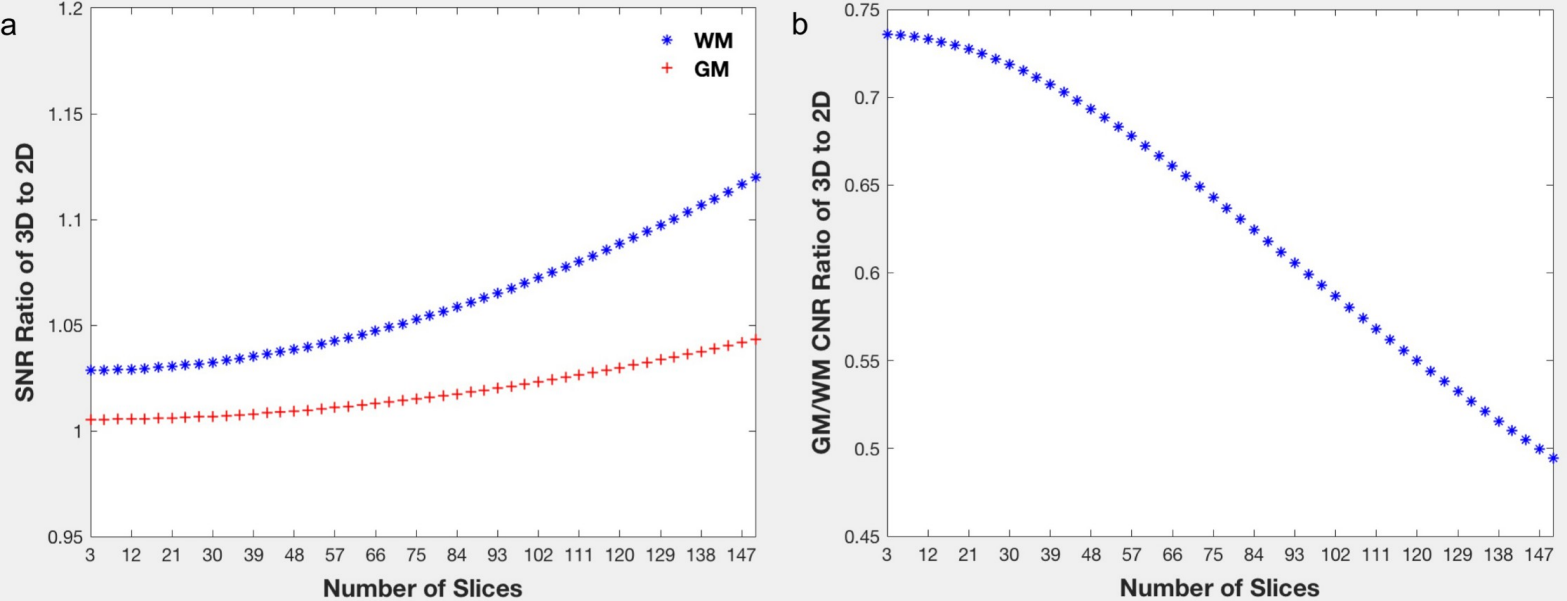
The simulation-derived (a) the SNR ratio of the 3D to the 2D SMS gradient-echo imaging in white and gray matter and (b) the GM/WM CNR ratio using Eqs (5) and (6), respectively, and parameters employed in current study. Note as the slice number increases in the simulation, the TR of 2D imaging also increases accordingly given it is a multiplanar interleaved acquisition.

The simulation of the 2D and 3D imaging showed a comparable SNR between the 2D SMS GRE and the 3D GRE imaging in both GM and WM (Fig 2A). Although the SNR of 3D GRE was still higher than that of 2D SMS GRE, it was only 1.12 times higher in WM and 1.04 times higher in GM even after the number of imaging slices reached 150. Imaging from healthy volunteers, however, showed significant differences in the SNRs between the 2D and 3D magnitude images in both WM (mean±standard deviation: 19.17±6.09 for 2D and 22.54±5.44 for 3D, *p* = 0.010) and GM (20.65±6.56 for 2D and 23.33±5.45 for 3D, *p* = 0.035), with 3D having a 17.6% higher SNR in WM and a 13.0% higher SNR in GM.

**Fig 3:**
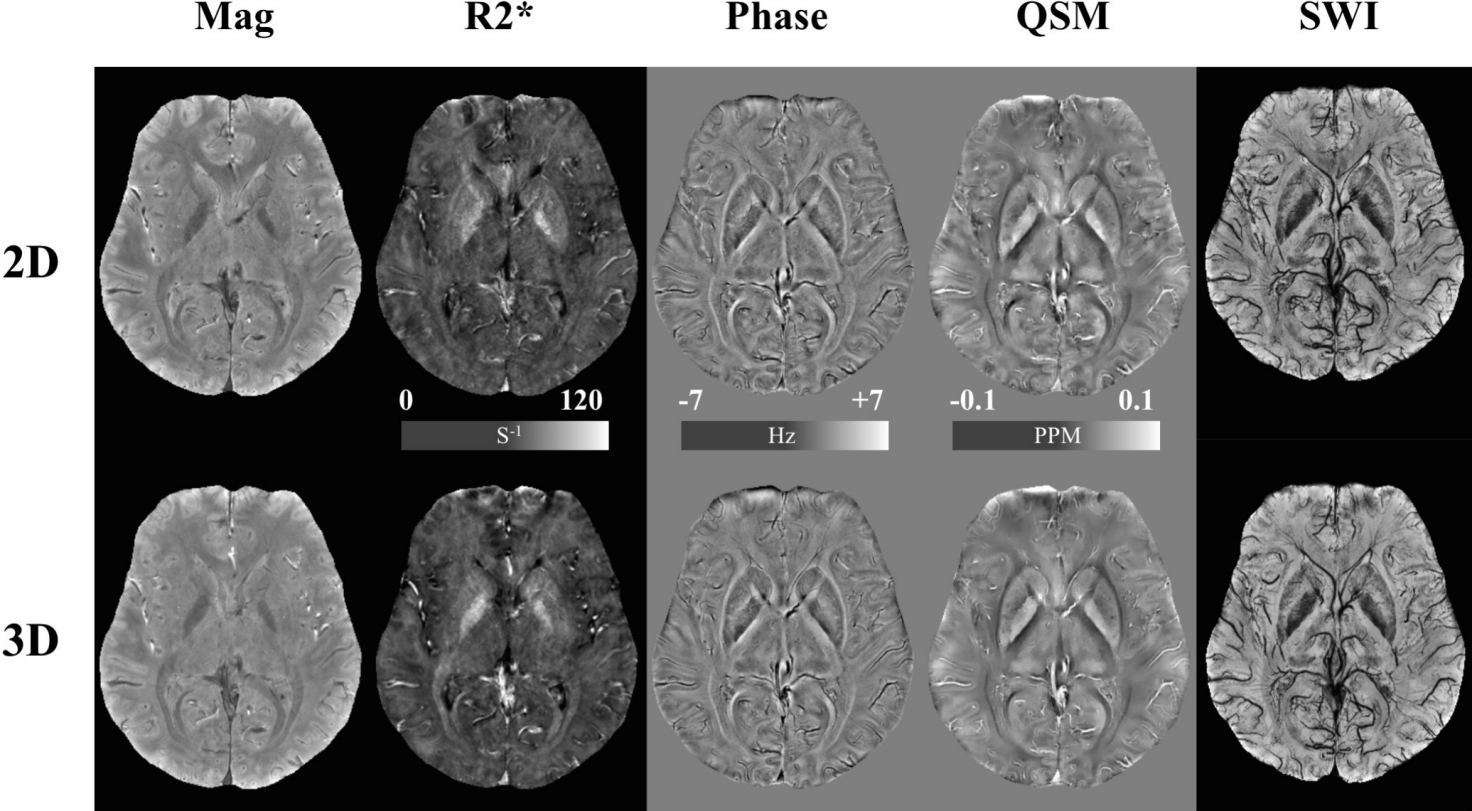
Images acquired using the 2D SMS gradient-echo (*top row*) and the 3D gradient-echo (*bottom row*) sequences in a volunteer.

**Fig 4:**
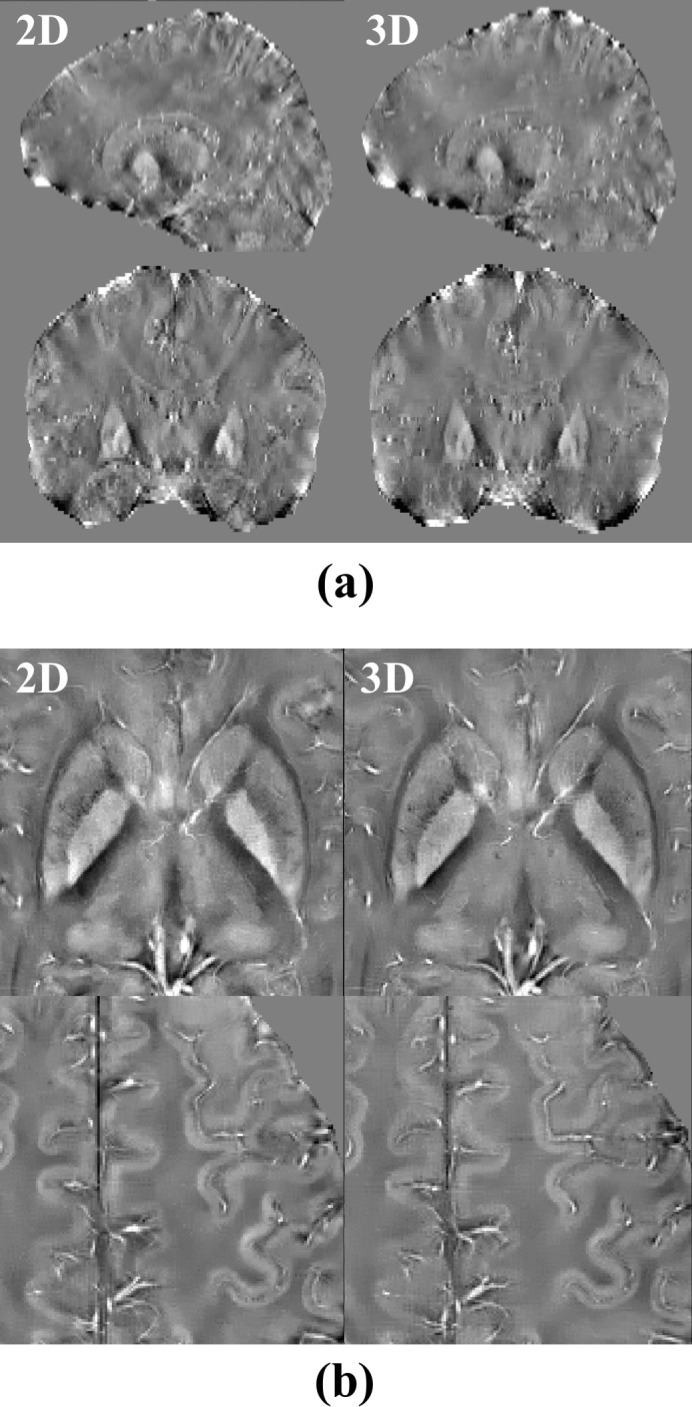
QSM images from a volunteer acquired using the 2D SMS gradient-echo (*left column*) and 3D gradient-echo (*right column*) sequences. (a) Neither the sagittal nor coronal images show streaking artifacts on either 2D or 3D images. (b) The 2D image shows a better contrast between globus pallidus and putamen than the 3D image (*top row*). Other deep gray matter (*top row*) and cortical gray/white matter (*bottom row*) show equal contrast on both the 2D and 3D images.

**Fig 5:**
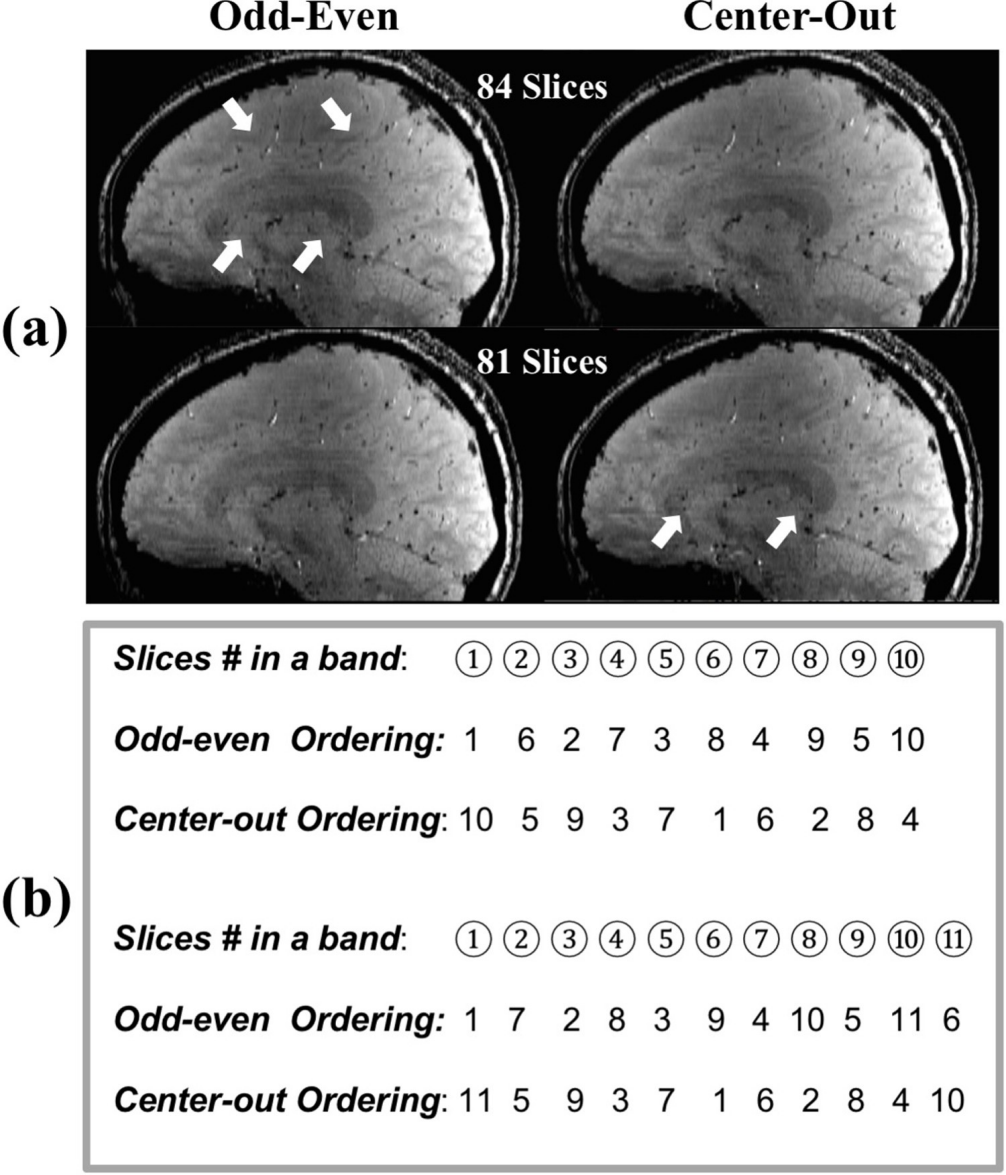
(a) 2D SMS (3 bands) gradient-echo magnitude images from one volunteer with a total slice of 84 (*top row*) and 81 (*bottom row*). The images were acquired using the odd-even slice ordering (*left column*) and center-out slice ordering (*right column*), respectively. Cross-talk artifacts (arrows) can be seen on the image acquired using the odd-even ordering when the slice number in a single band is even (84/3 = 28) and on the image acquired using the center-out ordering when slice number in a single band is odd (81/3 = 27). (b) An example used to illustrate the difference between the above two slice orderings, assuming 10 and 11 slices need to be scanned in a single band. Note, if the odd-even ordering is used, the slice 10 and its neighboring slice 1 in the next band will be excited back to back, resulting in cross-talk. This is not the case for the center-out ordering. However, when 11 slices need to be scanned, the situation is reversed, i.e., slices 10 and 11 in a same band will be excited back to back if the center-out ordering used, whereas no cross talk occurs if the odd-even ordering used.

On the other hand, the simulation also showed that the 2D imaging had a higher GM/WM CNR (Fig 2B). Moreover, the difference became larger as the imaging slice number increased. When 84 slices were imaged, the 2D imaging had a 1.60 times higher GM/WM CNR, which was close to the measurement from healthy volunteers, where the 2D imaging had a 1.89 times (87.5%) higher GM/WM CNR (1.49±0.64 for 2D and 0.79±0.48 for 3D, *p* = 0.002). Imaging from healthy volunteers also showed that the 2D imaging had a 17.3% and 13.3% higher CNR for GP/Pu (2.60±1.27 for 2D and 2.22±0.91 for 3D, *p* = 0.035) and GP/IC (4.18±0.93 for 2D and 3.69±0.84 for 3D, *p* = 0.021), respectively, on magnitude images (Fig 3). The 2D imaging also had a 24.6% higher CNR for GP/Pu (1.44±0.65 for 2D and 1.16±0.63 for 3D, *p* = 0.026) on QSM images (Fig 4). The 3D imaging had a significantly higher CNR only on R2* images for GP/Pu (2.24±1.95 for 2D and 3.15±1.52 for 3D, *p* = 0.010, Fig 3). No other significant differences in CNR were found (Table 2). In addition, close visual inspection of all images found no streaking artifacts on QSM images from 2D (as well as 3D) imaging (Fig 4). With an even/odd number of imaging slices, cross-talk was prevented with center-out/odd-even slice ordering, respectively (Fig 5).

**Table 2:** Contrast to noise ratios in different ROIs from 2D SMS and 3D imaging.

	Magnitude			Phase			R2*			QSM		
	2D	3D	*p* value	2D	3D	*p* value	2D	3D	*p* value	2D	3D	*p* value
**GP/Pu**	2.60 ±1.27^a^	2.22 ±0.91	**0.035**	0.47 ±0.32	0.39 ±0.29	0.438	2.24 ±1.95	3.15 ±1.52	**0.010**	1.44 ±0.65	1.16 ±0.63	**0.026**
**GP/IC**	4.18 ±0.93	3.69 ±0.84	**0.021**	3.22 ±0.53	3.07 ±0.97	0.614	5.91 ±2.52	6.63 ±2.10	0.118	4.50 ±1.09	4.21 ±0.64	0.297
**GM/WM**	1.49 ±0.64	0.79 ±0.48	**0.002**	3.15 ±0.70	3.00 ±0.89	0.667	0.48 ±0.35	0.60 ±0.48	0.477	2.72 ±1.47	3.29 ±1.31	0.308

^a^Mean ± Standard Deviation

CN = Caudate Nucleus; Pu = Putamen; GP = Globus Pallidus; IC = Internal Capsule; M = Gray Matter; WM = White Matter

**Fig 6:**
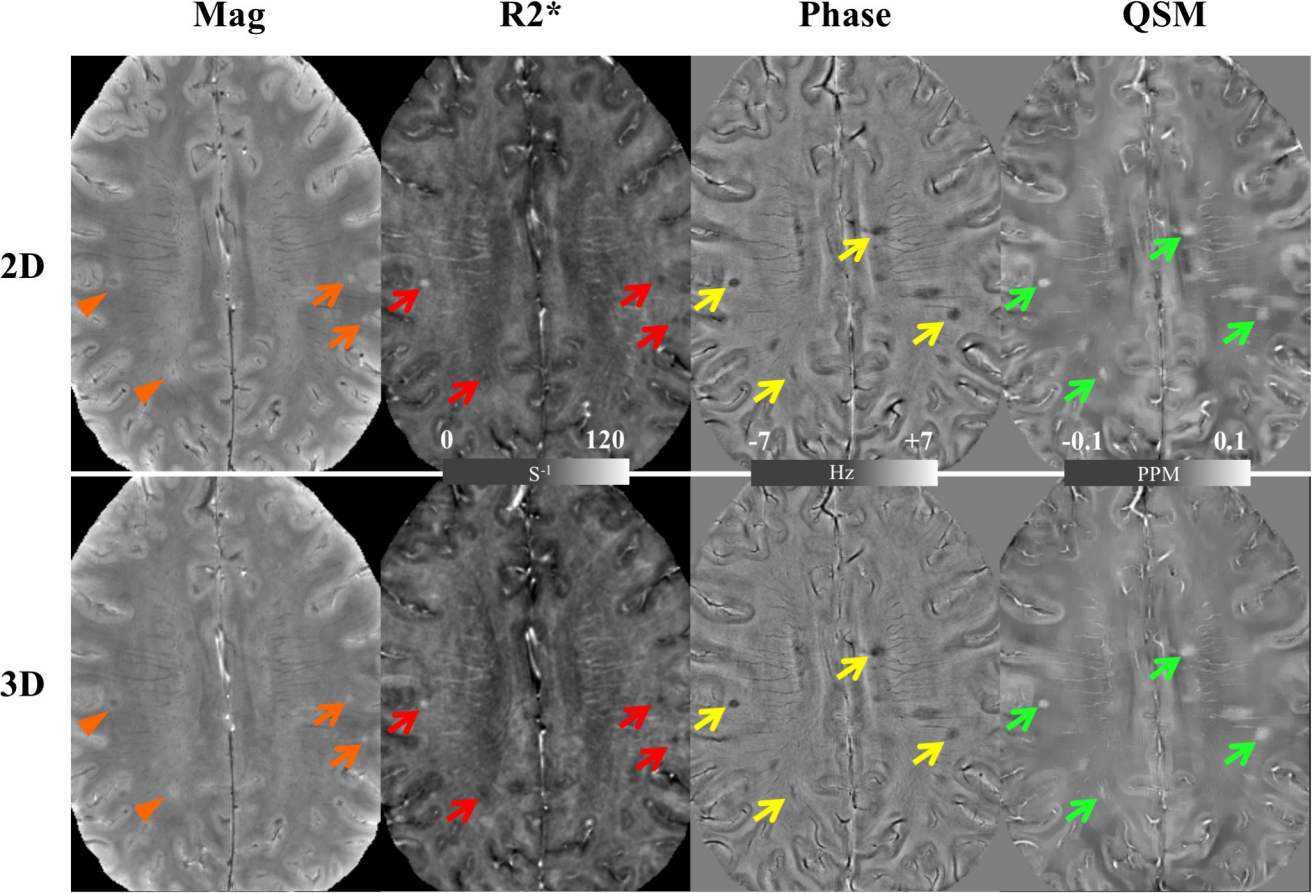
Images from the 2D SMS gradient-echo *(top row)* and 3D gradient-echo imaging *(bottom row)* from one MS patient. Overall MS lesions (with typical example highlighted with arrows) are visualized almost equally on the R_2_* (*red arrows*), phase (*yellow arrows*) and QSM (*green arrows*) images. On the magnitude images, two lesions (*orange arrows*) have similar visibility while the other two (*orange arrow head*) are better visualized on the 2D image. In addition, the 2D magnitude image shows a sharper edge between gray and white matter.

In our MS cohort, 132 lesions were identified on all imaging volumes from all MS patients. The lesion visibility rating showed a significantly higher visibility of MS lesions on all 2D images except R2* (Table 3 and Fig 6). In particular, 2D magnitude images had a 17.3% higher lesion visibility than 3D magnitude images (3.3±0.9 for 2D and 2.8±1.0 for 3D, *p*<10^−12^). It is also interesting to note that while all marked MS lesions in Fig 6 had similar frequency and susceptibility on phase and QSM images, the upper left one had a much higher R_2_* than other lesions on the R_2_* image, indicating different contrast mechanisms underlying these images.

**Table 3:** Mean rating score for the visibility^a^ of multiple sclerosis lesions.

	Magnitude	Phase	R2*	QSM
2D	3.3±0.9	2.6±1.3	2.6±1.3	3.0±1.1
3D	2.8±1.0	2.5±1.3	2.5±1.3	3.1±1.1
p value	<10−12	<10−4	<0.001	0.269

^a^Lesion visibility was rated by a 4-level scale, where 4 represents “*excellent*”: clearly visible with strong contrast between lesion and adjacent normal-appearing tissue; 3 represents “*good*”: visible with good contrast between lesion and adjacent normal-appearing tissue; 2 represents “*fair*”: faintly visible with limited contrast between lesion and normal-appearing tissue; 1 represents “*poor*”: not visible or barely visible with aid of corroborating sequences. Since any single lesion could visible or invisible on one or more image modalities, in our study the mean scores from different modalities were not comparable across modalities.

## Discussion

In this study, we developed a SMS acquisition with 2D GRE imaging to acquire susceptibility-contrast images after modifying the SMS calibration process. The process previously used in functional and diffusion MRI acquires a whole imaging volume separately to calibrate and unfold overlapped images due to SMS acquisition. While this approach is feasible for fMRI and DTI, where imaging volume need to be repeatedly scanned for multiple times, it is not feasible for 2D GRE imaging, where the imaging volume only needs to be scanned once. Although a calibration with coil sensitivity maps is possible [21], a separate scan is generally required, which may result in misregistration between scans. To avoid this additional scan, we designed an auto-calibration method that only samples a few central k-space lines for each slice and uses them as calibration data for image reconstruction. Our experiments showed that we were able to separate overlapped slices by sampling only 16 calibration lines from central k-space. Moreover, since these calibration lines can be recycled to calibrate in-plane parallel imaging, there is no additional cost on image reconstruction calibration when SMS imaging is performed alongside parallel imaging.

Another potential problem in SMS imaging is that slice numbers need to be odd within a single band to conform to traditional odd-even slice ordering so that cross-talk artifacts at the boundary of bands can be prevented [19]. To overcome this and make an even number of slices in a single band applicable in SMS imaging, we provide a center-out slice ordering option in our implementation to complement the odd-even ordering. With these two slice ordering options available, unnecessary extra slices in SMS imaging can be avoided.

Our experiment showed that the 2D SMS imaging had a lower SNR than the 3D imaging, whereas our simulation showed that the SNR of the two imaging methods was comparable. It is possible that the coil geometry factor does not fit our assumption and in fact differs between the 2D and 3D imaging. By formulating our 2D SMS acquisition equivalent to the 3D acquisition [38], both our 2D and 3D imaging have 6 times acceleration in 3D k-space. Even with the same effective acceleration factor, the two imaging methods still did not produce comparable SNRs, suggesting that there must be some other factors that could influence coil geometry signal losses, such as the number of calibration lines acquired, how aliasing or FOV shift is controlled during the image acquisition, the configuration of parallel imaging (3 x 2 for 3D, 2 x 3 for 2D) and the exact image reconstruction algorithm. Future work can analyze how much each individual factor impacts on coil geometry in order to understand the difference in the efficiency of the two different parallel imaging schemes used in our study, i.e., between the combination of SMS and in-plane acceleration in 2D imaging and parallel acquisition in both slice and in-plane directions in 3D imaging.

Although the 2D imaging had a lower SNR, it generated better gray/white matter, Globus pallidus/Putamen, and MS lesion contrast on the magnitude images. This is likely due to a much longer TR (over 30 times of that of 3D) used in the 2D imaging, which enhances both proton density and T_2_* weighting according to Eq 3, leading to better GM/WM contrast. This is also evident in our simulation (Fig 2B), where the 2D imaging gray/white matter CNR relative to that of the 3D imaging increased with the number of imaging slices. When slice number increases, the TR of the 3D imaging will not change, but the TR of the 2D imaging will increase because of the interleaved acquisition. While it is possible to increase the T2* weighting of the 3D imaging in the same way, this will clearly increase the 3D imaging time, which makes it less efficient compared to the 2D imaging. Other than the TR effect, motion induced blurring may further reduce image sharpness on the 3D images, as in general 3D imaging is more susceptible to motion blur by exciting a whole imaging volume instead of a single or several slices in 2D imaging. The improved gray/white matter and MS lesion contrast on our 2D magnitude image could add additional certainty in identifying correct location, hence a correct lesion type, of cortical MS lesions, in conjunction with conventional contrasts such as fluid attenuated inversion recovery (FLAIR) T_2_-weighted contrast. Since cortical gray matter lesions have been increasingly thought to play a more critical role in MS progression [39] and they are relatively difficult to identify at lower field strengths such as 3 or 1.5T [40–42], applying our sequence at a high field strength (e.g. 7T) may help in studying cortical lesion pathology in MS. This benefit, however, has to be evaluated on a larger cohort of patients to directly investigate gray matter lesions.

Finally, our study also suggests that a multi-echo sequence is preferred to generate R_2_* images simultaneously with other susceptibility-weighted images such as QSM to identify underlying pathological changes in MS lesions multi-parametrically. It has been shown that while both iron accumulation and demyelination increase QSM, the former increases R2* and the latter decreases R2* [4,12,43]. With different contrast mechanisms underlying R2* and QSM, one could explain why all marked MS lesions in Fig 6 had similar frequency and susceptibility on phase and QSM images, but the upper left lesion had a much higher R_2_* than other lesions on the R_2_* image. In this particular lesion, iron accumulation presumably dominated over demyelination, whereas in the other lesions demyelination dominated over iron accumulation.

## Conclusions

As an alternative to 3D GRE imaging, a multi-echo 2D GRE imaging with simultaneous multi-slice acquisition can be employed to perform susceptibility contrast imaging and provide T_2_*-weighted magnitude, phase, SWI, R_2_* and QSM images, with matched scan time and image resolution. While this multi-echo 2D imaging can have a slightly lower SNR on magnitude images compared to the 3D imaging, it can generate better gray/white matter and MS lesion contrast on both magnitude and QSM images. In addition, 2D imaging is likely to be more robust to motion blurring compared to 3D imaging. We anticipate it will be useful in the diagnosis of neurodegenerative diseases, where information from MR susceptibility contrast has the potential to become an important biomarker.

## Supporting information

S1 File Volunteer demographic information. (XLSX)

S2 File Multiple sclerosis patient clinical information. (XLSX)

S3 File Signal to noise ratio measured for volunteers. (XLSX)

S4 File Contrast to noise ratio measured for volunteers. (XLSX)

S5 File Multiple sclerosis lesion visibility rating for patients. (XLSX)
